# The impact of silence pedagogy on vulnerable students’ engagement in physical education: a mini-review

**DOI:** 10.3389/fpsyg.2026.1803241

**Published:** 2026-04-20

**Authors:** Qing Deng, Qing Jun Wang, Xue Feng Wang, Xiang Qian Mou

**Affiliations:** 1 School of Sports Science and Physical Education, Nanjing Normal University, Nanjing, China; 2 School of Physical Education, Nanjing Xiaozhuang University, Nanjing, China; 3 School of Journalism and Communication, Nanjing Normal University, Nanjing, China

**Keywords:** behavioral engagement, cognitive engagement, emotional engagement, physical education, silence pedagogy, vulnerable students

## Abstract

In recent years, improving vulnerable students’ engagement in the classroom has received increasing attention from researchers. Existing literature indicates that vulnerable students with low levels of classroom engagement often show signs of confused thinking, distracted attention, and insufficient learning investment. This phenomenon has been widely discussed among researchers in the field of education. To improve classroom engagement among vulnerable students, researchers have proposed various interventions. One of these interventions is the implementation of silence pedagogy in the classroom. However, existing research on silence pedagogy has mainly focused on mathematics and foreign language classrooms, whereas research in physical education (PE) remains limited. This raises questions about what silence pedagogy is and how it affects vulnerable students’ engagement in PE classes. Drawing on the Preferred Reporting Items for Systematic Reviews and Meta-Analyses (PRISMA) search strategy, we conducted a systematic search of databases such as the Web of Science and Scopus and included 21 articles. We then reviewed these articles and examined the definition of silence pedagogy and its impact on students’ engagement. The analysis revealed that silence pedagogy could enhance vulnerable students’ cognitive, behavioral, and emotional engagement. This study extends the existing knowledge base on silence pedagogy by reviewing its effects on classroom engagement among vulnerable students in PE contexts.

## 1 Introduction

In the Analects of Confucius (Confucius, 2016), there is a well-known statement:

*“They who know the truth are not equal to those who love it, and they who love it are not equal to those who delight in it.” (p. 24)*

Confucius was a great educator, thinker, and the founder of Confucianism in ancient China. This statement presents three distinct levels of learning attainment: Learning knowledge, loving knowledge, and deriving joy from knowledge (Confucius, 2016). In other words, when students engage in learning knowledge, they can develop a love of learning (interest) and attain joy. In general, Confucius’s point reminds us that it is crucial to use effective pedagogy to enhance students’ engagement, as it can cultivate students’ interest in learning and enable them to derive joy from knowledge. This, in turn, may contribute to students’ continued pursuit of “learning opportunities” and “self-initiated learning” (Barron, 2006, p. 193). This raises an important question: Which pedagogical approaches can be employed to increase vulnerable students’ engagement in the classroom? In this article, we introduce a teaching technique known as silence pedagogy, which may increase vulnerable students’ engagement.

In recent years, improving vulnerable students’ engagement in the classroom has received increasing attention from researchers. Existing literature indicates that vulnerable students with low engagement usually experience “discouragement and disorganization,” “distraction,” and “disinterest” in classes (Ramos et al., 2020, p. 8388). This could lead to “dropout” and “graduation rates falling” (Perry, 2022, p. 1313). To improve classroom engagement among vulnerable students, researchers have proposed various interventions (Ha and Li, 2014; Konicarova, 2014; Matatyaho, 2015). One of these interventions is the implementation of silence pedagogy in the classroom.

For example, the study by Matatyaho (2015) aimed to assess whether “1 minute of silence” can decrease anxiety and improve math test scores among students with disabilities (p. 1). The author found that silence could “decrease anxiety” (p. 134), enhance “self-efficacy” (p. 127), and “improve math test scores” (p. 134) among students with disabilities, which may, in turn, promote their classroom engagement. In foreign language classes, Konicarova (2014) also noted that the “Silent Way” [sic] could “facilitate students’ self-correction activities” and “develop self-reflection” (p. 65). In general, by reviewing prior studies, we found that silence pedagogy may improve classroom engagement among vulnerable students in mathematics and foreign language classrooms.

However, few review articles have examined silence pedagogy in physical education (PE) contexts. For instance, authors such as Renau (2016) and Konicarova (2014) have provided an overview of the effects of silence pedagogy on foreign language learners’ engagement. Other researchers have also found that silence pedagogy in mathematics classrooms facilitates students’ cognitive engagement (Lee and Sriraman, 2013) and reduces mathematics anxiety (Matatyaho, 2015). Therefore, it remains unclear whether silence pedagogy can also enhance classroom engagement among vulnerable students in PE contexts.

Recently, Su et al. (2023) called on future researchers to “reconceptualise and reclaim the place of silence in education” (p. 40). Therefore, in response to this call, this article aims to synthesize research on silence pedagogy and discuss its potential effects on students’ classroom engagement in PE contexts.

To achieve these objectives, this research is guided by the following two questions: What is silence pedagogy, and what are the impacts of silence pedagogy on vulnerable students’ engagement in PE classes? By answering these two questions, we can better understand the definition of silence pedagogy. With this understanding, researchers can better identify the gaps in the field of pedagogy and develop targeted research questions. Teachers can also gain insight into how to use it to support vulnerable students. This study extends the existing knowledge base by reviewing the effects of silence pedagogy on vulnerable students’ engagement in PE contexts.

This article is divided into four sections. The first section defines the core terms. The second section describes the methodology of the study. The third section presents and discusses the research questions and main findings of the study. The fourth section presents the conclusions and limitations and provides recommendations for further research.

## 2 Definition of terms

Before data collection, it is crucial to define the terms related to the research question, as this can help reduce the risk of false-positive results to some extent (Barrot, 2023). That is, literature unrelated to the research topic was inadvertently included. According to Van Kamp and Davies (2013), a vulnerable group is susceptible to “physical or emotional injury or attack” (p. 153). In our article, vulnerable groups are conceptualized as students with disabilities, children with attention deficit hyperactivity disorder (ADHD), and other vulnerable students. Students’ engagement is defined as a “multidimensional construct” that involves cognitive, behavioral, and emotional dimensions (Fredricks, 2011, p. 328). Cognitive engagement can be defined as being willing to exert effort to master ideas and skills (Fredricks, 2011). Behavioral engagement can be defined as participation and involvement in academic tasks and activities, and emotional engagement can be defined as positive and negative reactions (Fredricks, 2011).

## 3 Methodology

### 3.1 Types/sources of data

#### 3.1.1 Search strategy

The process of article selection drew on the Preferred Reporting Items for Systematic Reviews and Meta-Analyses (PRISMA) search strategy (Page et al., 2021). On 5th August 2025, we searched Scopus and the Web of Science for peer-reviewed articles related to silence pedagogy, vulnerable groups, and engagement. We operationalized different permutations of each keyword based on previously validated searches. For silence pedagogy, we followed Ollin’s (2008) search terms, and for vulnerable groups, we followed Limantė and Теrеškinas’s (2022) search terms. For engagement, we drew on several studies to identify keyword variants (Karimah and Hasegawa, 2022; Xuemei et al., 2023). In short, this made us more confident that we would capture appropriate citations in the searches.

We applied the title and abstract fields in the search. The full details are provided in Table 1. Our initial search identified a total of 420 articles in the Web of Science and 404 articles in Scopus, which were imported into Zotero reference management software. Of these 824 articles, 42 were identified as duplicates, leaving a total of 782 for the screening and eligibility stages.

**Table 1:** Search terms used in the Web of Science and Scopus for studies related to silence pedagogy, vulnerable groups, and engagement in PE classrooms.

Step	Terms	Results
Database: Web of science		
1	TS = (“Organizational silence”OR “Organisational voice” OR “Silence behavior” OR “Prohibitive voice” OR “silence culture” OR “Quiescent Silence” OR “Acquiescent Silence” OR “Opportunistic Silence” OR “Safety Silence” OR “keeping silent at work” OR “silence” OR “silences” OR “silent” OR “listening” OR “silent practice” OR “voices behind the silence” OR “saying nothing” OR “Silence” OR “unspoken”)	106,105
2	TS = (“pedagogy” OR “pedagogies” OR “Teacher Practice” OR “education” OR “instruction” OR “pedagogical approaches” OR “study” OR “pedagogical”)	15,982,467
3	TS = (“physical education” OR “sport education” OR “physical activity” OR “motor performance” OR “motor skill” OR “motor ability” OR “motor competence” OR “athletic participation” OR “exercise” OR “school” OR “during classroom lessons”)	1,020,130
4	TS = (“participation” OR “expression” OR “engagement” OR “involvement” OR “cognition” OR “cognitive activities” OR “executive function”OR “executive control” OR “working memory” OR “conduct” OR “behavior” OR “movement” OR “Emotion” OR “emotional” OR “interaction” OR “practice” OR “internal behavioral engagement”)	10,807,744
5	TS = (“Vulnerable group” OR “marginalised groups” OR “groups at risk” OR “groups of social exclusion” OR “single parents” OR “minorities” OR “immigrants” OR “health loss” OR “health impair” OR “people at risk” OR “stigma participants” OR “gatekeeper” OR “low-SES students” OR “student” OR “female students” OR “transgender” OR “disabled” OR “developmental disability” OR “intellectual disability” OR “student with single parents” OR “minorities” OR “people with stress” OR “people with anxiety” OR “people with emotional disturbances” OR “the disempowered”)	428,976
6	1 AND 2 AND 3 AND 4 AND 5	420
Database: Scopus		
1	TITLE-ABS-KEY(“Organizational silence”OR “Organisational voice” OR “Silence behavior” OR “Prohibitive voice” OR “silence culture” OR “Quiescent Silence” OR “Acquiescent Silence” OR “Opportunistic Silence” OR “Safety Silence” OR “keeping silent at work” OR “silence” OR “silences” OR “silent” OR “listening” OR “silent practice” OR “voices behind the silence” OR “saying nothing” OR “Silence” OR “unspoken” OR “different forms of silence”)	214,748
2	TITLE-ABS-KEY(“pedagogy” OR “pedagogies” OR “Teacher Practice” OR “education” OR “instruction” OR “pedagogical approaches” OR “study” OR “pedagogical”)	38,632,464
3	TITLE-ABS-KEY(“physical education” OR “sport education” OR “physical activity” OR “motor performance” OR “motor skill” OR “motor ability” OR “motor competence” OR “athletic participation” OR “exercise”)	
4	TITLE-ABS-KEY(“participation” OR “expression” OR “engagement” OR “involvement” OR “cognition” OR “cognitive activities” OR “executive function”OR “executive control” OR “working memory” OR “conduct” OR “behavior” OR “movement” OR “Emotion” OR “emotional” OR “interaction” OR “practice” OR “internal behavioral engagement”)	22,311,339
5	TITLE-ABS-KEY(“Vulnerable group” OR “marginalised groups” OR “groups at risk” OR “groups of social exclusion” OR “single parents” OR “minorities” OR “immigrants” OR “health loss” OR “health impair” OR “people at risk” OR “stigma participants” OR “gatekeeper” OR “low-SES students” OR “student” OR “female students” OR “transgender” OR “disabled” OR “developmental disability” OR “intellectual disability” OR “student with single parents” OR “minorities” OR “people with stress” OR “people with anxiety” OR “people with emotional disturbances” OR “the disempowered”)	2,482,188
6	1 AND 2 AND 3 AND 4 AND 5	404

#### 3.1.2 Inclusion and exclusion criteria

We applied a series of inclusion and exclusion criteria. Articles were included if they: (i) were written in English; (ii) were published in a peer-reviewed journal; (iii) were qualitative, quantitative, or mixed methods research articles; and (iv) involved research participants who were vulnerable students. Articles were excluded if they were review articles, opinion articles, or did not focus on learning engagement, as these types of studies were not relevant to our research questions.

Of the 501 records screened, 220 were excluded because they were book chapters, conference papers, or not in English, leaving 281 articles for retrieval. We were able to obtain the full text of all 281 articles for eligibility assessment. After reviewing the full texts, we excluded 252 articles that were not relevant to our research questions. In addition, 16 review articles and opinion articles were excluded based on document type. We also identified an additional eight articles by scanning the reference list, following peers’ recommendations, and using Google Scholar. Among these, a doctoral dissertation and a review article were included because they were relevant to our research topic. This resulted in a final review sample of 21 articles for data analysis.

#### 3.1.3 Extraction and analysis

Our procedure for data extraction and analysis followed the steps proposed by Yang and Whatman (2025): (1) Saved the 21 articles in a folder; (2) read these articles; and (3) extracted the main study parameters into a Microsoft Excel literature grid with multiple tabs. Data included author(s)/year of publication, country of study, study design, study participants, data collection approach, analysis methods, and results/findings. Descriptive information for the 21 articles is provided in Table 2.

**Table 2:** Extraction of descriptive information from the 21 included articles.

Number	Authors	Geographical location	Title	Journal	Study design	Research participants	Data collection method	Data analysis techniques	Results/findings	Relevancy to research
Publication 1	Fisette (2013)	USA	‘Are you listening?’: adolescent girls voice how they negotiate self- identified barriers to their success and survival in physical education	Physical Education and Sport Pedagogy	Qualitative research	Seven 9th and 10th grade girls	Group interviews, descriptive field notes, and informal conversations	Coding and the constant comparative method	Individual lived experiences show how participants embody comfort and enjoyment by navigating their participation within particular contexts	High
Publication 2	Cardiff et al. (2023)	Ireland	‘Just let them have a say!’ Students’ perspective of student voice pedagogies in primary physical education	Irish Educational Studies	Qualitative research	39 students (10–11 years old) and a teacher	Reflections transcripts/scrapbooks/focus group interviews	Thematic analysis	Students reported that: My voice counts/I can use my voice to make choices that matter to me/Taking control and getting to be in charge	High
Publication 3	Munk and Agergaard (2018)	Denmark	Listening to students’ silences – a case study examining students’ participation and non-participation in physical education	Physical Education and Sport Pedagogy	Qualitative research	24 students (7th–9th grades), including 13 girls	Focus group interviews, PE class observations, and descriptive field notes	Thematic analysis	Teachers unintentionally transmit values and attitudes that affect PE experiences; socially less respected students often do not participate	High
Publication 4	Littlefair et al. (2025)	England	Pupil voice in physical education and the desire for (in)visibility	SPORT, EDUCATION AND SOCIETY	Qualitative research	154 pupils (14–16 years old)	Focus groups, questionnaire surveys, and interviews	Thematic analysis	Students identified as ‘low ability’ suggested giving choices of activities and class groupings to increase engagement in sport	High
Publication 5	Oliver and Oesterreich (2013)	USA	Student-centered inquiry as curriculum as a model for field-based teacher education	J. CURRICULUM STUDIES	Qualitative research	25 freshman students and 11 teachers	Field notes, observations, researchers’ notes, and focus group interviews	Grounded theory (open, axial, and selective coding)	Promotes lifelong physical activity and facilitates interests, motivation, and learning	High
Publication 6	Howley and O’Sullivan (2021)	Ireland	‘Getting better bit by bit’: Exploring learners’ enactments of student voice in physical education	Donal Howley	Qualitative research	18 students aged 13–14 years and teachers aged 25–26 years	Focus group interviews	Inductive thematic analysis	Enhances students’ democratic experiences and influences decision-making, program content, levels of activity, and participation	High
Publication 7	Brooker and Macdonald (1999)	Australia	Did we hear you?: Issues of student voice in a curriculum innovation	Journal of Curriculum Studies	Mixed methods research	27 teachers and 822 students (years 11–12)	Audio-taped interviews, field notes, small group interviews, and surveys	Grounded approach incorporating demographic analysis	Findings informed curriculum design	Medium
Publication 8	Lyngstad et al. (2016)	Norway	Understanding pupils’ hiding techniques in physical education	Sport, Education and Society	Qualitative research	Six PE teachers and eight former pupils	Focus groups, retrospective interviews, and observations	Coding and interpretation following hermeneutic principles	Students attempted to downplay and avoid problems	High
Publication 9	Lee and Sriraman (2013)	Korea	An Eastern Learning Paradox: Paradoxes in Two Korean Mathematics Teachers’ Pedagogy of Silence in the Classroom	Interchange	Mixed methods research	32 middle school teachers, including two female teachers	Investigation, semi-structured interviews, field notes, and video recordings	Coding using grounded theory	Teachers’ approaches attracted students’ attention and facilitated constructivist learning through silence	High
Publication 10	Ha and Li (2014)	Australia	Silence as right, choice, resistance and strategy among Chinese ‘Me Generation’ students: implications for pedagogy	Discourse: Studies in the Cultural Politics of Education	Qualitative research	Four Chinese MG students	Semi-structured, in-depth interviews and one-to-one conversations	Not Explicitly Mentioned	Interpretations of silence were used to promote learning in context	High
Publication 11	Mahoney et al. (2022)	USA	Springsteen-omics: contemplative pedagogy and Springsteen in undergraduate economics courses	The Journal of Economic Education	Mixed methods research	Undergraduate students	Observation notes and learning reflections(LRs)	Thematic analysis, coding, quantifying responses, and narrative analysis	Students gained an appreciation for economics, deepened their ability to identify and describe lyrical intent, and developed a more nuanced understanding of the economy	Medium
Publication 12	Ollin (2008)	United Kingdom	Silent pedagogy and rethinking classroom practice: structuring teaching through silence rather than talk	Cambridge Journal of Education	Qualitative research	25 teachers	Detailed interviews, face-to-face interviews, detailed semi-structured interviews, and open-structured interviews	Not Explicitly Mentioned	Provided slow-time for cognitive processing, relaxation, absorption of prior content, and opportunities for reflection	Medium
Publication 13	Rowe (1974)	USA	Pausing Phenomena: Influence on the Quality of Instruction	Journal of Psycholinguistic Research	Qualitative research	16 teachers and their students	Observations and tape recordings	Coding	Increasing the length of response time leads to a higher incidence of speculative responses, more evidence–inference statements, and greater variety in types of verbal moves	Medium
Publication 14	Hanna (2021)	United Kingdom	Silence at school: Uses and experiences of silence in pedagogy at a secondary school	British Educational Research Journal	Qualitative research	20 teachers and 35 students aged 14–15 years	Group discussions and interviews	Coding	Silence was used to enhance learning productivity and to promote respect in the classroom	High
Publication 15	Scudder (2024)	USA	The art of listening in the age of AI	Journal of International Political Theory	Qualitative research	University students	Watching and experiential observations	Not Explicitly Mentioned	Teachers focused on listening to understand students and improve their learning	Medium
Publication 16	Shen (2025)	China	Analysis of Classroom Interaction Efficacy of English Teachers in Higher Education and Modeling Research Based on Multiple Regression	Journal of Combinatorial Mathematics and Combinatorial Computing	Mixed methods research	Third-year university English students and their teachers	Observation, investigation, and videotaping	Coding, ratio analysis, and matrix analysis	A reasonable structure of teacher–student language ratio was identified	High
Publication 17	Hanna (2022)	United Kingdom	Seen and not heard: Students’ uses and experiences of silence in school relationships at a secondary school	Sage Journals	Qualitative research	42 young people and 27 teachers	Consultations, conceptual group discussions, and semi-structured interviews	Inductive coding	Students use silence to negotiate their perceptions and expectations of respect, resist teacher authority, and preserve their dignity	Medium
Publication 18	Tan et al. (2025)	Singapore	Breaking the silence: understanding teachers’ use of silence in classrooms	Pedagogies: An International Journal	Qualitative research	33 Grade 9 students and two teachers	Video recordings and eye-tracking	Coding and event-oriented inquiry	Silence was used during activities such as students copying notes while teacher checked attendance, distributed worksheets, or searched for teaching resources	Medium
Publication 19	Matatyaho (2015)	USA	Silence Improves Anxiety Levels and Test Scores Among Children With Disabilities	Doctoral dissertation of Walden University	Quantitative research	55 students with special needs	Convenience sampling	Statistical significance	Students with lower levels of anxiety achieved higher test scores	High
Publication 20	Yousefizadeh (2025)	Iran	Teaching English to Students with ADHD: Delving into Possibilities and Challenges through a Qualitative Lens	Qualitative Inquiry as Praxis in L2 Studies	Quantitative research	A male student with ADHD	Field notes, daily observations, note-taking, and video recordings	Constant comparative analysis	Minimal teacher interference combined with maximizing student production was particularly effective	High
Publication 21	Konicarova (2014)	Czech Republic	Psychological principles of learning in children with ADHD and dyslexia	Activitas Nervosa Superior	Review article	Children with ADHD and dyslexia	Not explicitly mentioned	Not explicitly mentioned	Facilitates students’ self-correction activities and helps them develop self-reflection and awareness of their own learning	High

## 4 Findings and discussion

### 4.1 Research question 1: what is silence pedagogy?

In the 21 included articles, some researchers described silence pedagogy as “wait time” (Rowe, 1974, p. 203), “pauses” (Tan et al., 2025, p. 341), and “blank space” (Hanna, 2021, p. 1170). Others examined the roles of silence pedagogy in classroom settings (Cardiff et al., 2023; Rowe, 1974; Tan et al., 2025). For example, Tan et al. (2025) suggested that teachers used silence pedagogy to allow students to take notes.

In general, the included articles highlight the following claims regarding the definition and role of silence pedagogy:

1 Silence pedagogy can be defined as a teaching technique that teachers employ to facilitate students’ learning. It can be applied during lesson preparation, classroom interactions, and other classroom activities.
2 Teachers often use silence pedagogy in the classroom to enhance students’ cognitive, behavioral, and emotional engagement.

Next, we illustrate each claim by referencing the included articles. With respect to the first knowledge claim, some authors, such as Ollin (2008), Rowe (1974), and Tan et al. (2025), provided descriptions of teacher silence in the classroom. For example, the study by Ollin (2008) reported that teachers used various types of silence for teaching. The author found that silence can be used “as a tool to achieve particular aims and to aid learning” (Ollin, 2008, p. 276). According to the author, teachers used silence to give students “time to think” what they have learned (Ollin, 2008, p. 276). As Ollin (2008) wrote,

Silence was also described as being used as a relaxation/slowing down time either at the beginning, to settle down the group, or at the end, to enable them to absorb what had gone before. Teachers also identified its use for focus, discipline and control and for thinking time. (p. 270)

Ollin’s point here suggests that silence pedagogy could serve as a teaching technique to support teachers’ teaching and students’ learning (knowledge claim 1).

The study by Tan et al. (2025) provides a second example. Their study focused on the impact of teachers’ use of silence on classroom instruction. The authors used cameras to capture teachers’ silent behaviors, such as fixation. Tan et al. (2025) noted that “silence was used to check students’ attendance and distribute worksheets or search for teaching resources” in class (p. 337). This observation is valuable, as it shows that silence pedagogy could serve as a teaching technique to assist teachers in preparing classroom activities and materials (knowledge claim 1). Tan et al. (2025) further noted that teachers’ use of silence could provide students with time to “copy notes” (p. 339) and for “reflection and thinking” (p. 340). In this way, silence pedagogy allows students to review and reflect on the knowledge they have learned. Overall, Ollin (2008) and Tan et al. (2025) illustrated that silence pedagogy could be used as a teaching technique to support teachers’ teaching and students’ learning.

With respect to the second knowledge claim, silence pedagogy could enhance students’ cognitive, behavioral, and emotional engagement. For instance, Rowe (1974) examined the impact of teachers’ “wait time” following a question on children’s language and logical development (p. 203). The researcher analyzed classroom tape recordings on teachers’ wait time. This study found that students’ responses changed when the wait time was prolonged. Rowe (1974) noted that when the wait time reached 3–5 s, there was an increase in students’ verbal moves, the incidence of speculative responses, and the number of evidence-inference statements. In other words, during this wait time, students could synthesize knowledge in their heads. This aligns with Ollin’s (2008) point that teachers’ silence in teaching can provide students with “time to think” (p. 273) (knowledge claim 2). Similarly, Rowe (1974) noted that “time is bought for more cognitive processing” (p. 223). Therefore, we believe that this period of silence is an important condition for promoting students’ cognitive, behavioral, and emotional engagement.

Notably, the study by Rowe (1974) had several and limitations. One such limitation was the use of a single data source—classroom tape recordings—which might have affected the credibility of the research (Yang and Whatman, 2025). According to Owen and Rogers (2011), well-known evaluators, assembling information should be based on various sensory inputs. Thus, researchers can also observe the behaviors of students in the classroom through the video recordings. In addition, teachers, either as full participants or observers, can take notes during the implementation of silence pedagogy.

To sum up, silence pedagogy is defined as a teaching technique that supports teaching and enhances students’ engagement. This approach can be applied during lesson preparation, classroom interactions, and other classroom activities. Next, we will discuss the impact of silence pedagogy on vulnerable students’ engagement in PE classes.

### 4.2 Research question 2: what is the impact of silence pedagogy on vulnerable students’ engagement in PE classes?

By reading the included articles, we found that in PE classes. It is necessary to acknowledge that, in PE classes, studies on the use of silence pedagogy for vulnerable students have received limited attention. This observation is in line with our expectations. As Hanna (2022) noted, when teachers remain silent in the classroom, it may imply a deficit in teacher authority. However, in physical education contexts, teachers are typically expected to actively exercise instructional authority (Lyngstad, 2017). This emphasis on visible instructional authority suggests that silence is rarely positioned as a pedagogical strategy.

Nevertheless, some studies have examined the impact of silence pedagogy on vulnerable students’ engagement in other disciplinary contexts (Konicarova, 2014; Matatyaho, 2015; Yousefizadeh, 2025). We discuss these studies here to further highlight their contributions to the literature.

For example, the study by Konicarova (2014) focused on the role of the silent way in language learning for children with ADHD and dyslexia. The author noted that teachers used the silent way and simple linguistic tasks to empower children to produce examples of the new language. This approach is a discovery-based activity that contributes to students’ cognitive and behavioral engagement. For example, Konicarova (2014) further explained that, by using this method, the teacher could facilitate “students’ self-correction activities” and help them “develop self-reflection and awareness about their own learning” (p. 65). In this process, the learner was described as “a principal actor” (Konicarova, 2014, p. 65). In general, the silence technique could facilitate students’ cognitive and behavioral engagement.

Yousefizadeh (2025) provides a second example. The author aimed to identify the challenges faced by a male ADHD student in foreign language learning and to evaluate effective teaching methods. The author tracked the student’s learning progress over a semester through daily observations, note-taking, and video recordings. The study found that there were challenges, but adaptive student-centered strategies—such as the “silent way”—can improve learning outcomes (Yousefizadeh, 2025, p. 4). Specifically, it could contribute to “reducing anxiety” (p. 13) and “fostering self-guided learning” for the student (Yousefizadeh, 2025, p. 14). By reading this article, we also found that this approach can help him complete learning tasks (Yousefizadeh, 2025). In short, this approach could be an effective tool for enhancing classroom engagement in foreign language classes.

Although this study has many merits, it also has some methodological limitations. For example, in the author’s study, all data were derived from a single observer (Yousefizadeh, 2025), which may result in incomplete information and undermine the credibility of the observations. As Denzin (1973) noted, investigator triangulation involves using more than one observer to increase the credibility of observations. In this case, the author could have collected information not only from the student but also from other teachers and students.

The third example is the study by Matatyaho (2015), which focused on the use of one minute of silence in mathematics classes to improve the learning of students with disabilities. The author compared two groups’ levels of anxiety and math academic scores: One experiencing no silence and the other experiencing one minute of silence. The study concluded that the silence technique contributed to “increasing positive healthy behavior” (p. 132) and “lowering anxiety levels while increasing math scores” (Matatyaho, 2015, p. 134). In other words, silence pedagogy can promote students’ positive performance and enhance their engagement.

Overall, the reviewed literature suggests that silence pedagogy can serve as an important tool for enhancing vulnerable students’ cognitive, behavioral, and emotional engagement. To the best of our knowledge, in the English-language literature on silence pedagogy, no study has explored the impact of this pedagogy on vulnerable students’ engagement in PE classes. In contrast, research in mathematics and language classes has shown that silence pedagogy can promote vulnerable students’ engagement. These articles contribute significantly to our knowledge.

## 5 Conclusion and limitation

This article aims to respond to Su et al.’s (2023) call to re-conceptualize and reclaim the role of silence in education. We describe the definition of silence pedagogy and explore its impact on vulnerable students’ engagement in PE classes. Specifically, silence pedagogy can be defined as a teaching technique that teachers employ to facilitate students’ learning in the classroom. This approach may contribute to enhancing vulnerable students’ cognitive, behavioral, and emotional engagement. Teachers can use silence pedagogy to help vulnerable students reduce anxiety and foster self-directed learning.

We acknowledge a limitation of this study. Specifically, we only searched two databases, which may have restricted the inclusion of relevant literature available in other sources. Given this limitation, researchers should exercise caution when interpreting the findings of this study and avoid overgeneralizing the study’s conclusions. Despite this limitation, we implemented several measures to enhance the transparency of our research findings. For example, we selected search terms based on prior literature (Karimah and Hasegawa, 2022; Limantė and Теrеškinas, 2022; Ollin, 2008; Xuemei et al., 2023). This approach gave us confidence in retrieving relevant studies. In addition, we provided detailed information for the 21 included articles, including the topic, study design, and findings/results, to further enhance the transparency of our review. Future research could review literature from additional databases such as EBSCO, Springer, and PubMed.

In addition, future research could focus on the impact of silence pedagogy on students in PE classes and on developing assessment criteria for students’ engagement, which is a valuable research topic. Specifically, according to Griffin (2017) and Yang and Whatman (2025), researchers can follow the evaluation logic and steps to assess students’ engagement. To better assess the state of the evaluand, researchers can also draw from the study by Wilson (2023) and design a hypothetical construct map to determine the position of the evaluand. In this process, if teachers observe a decline in students’ engagement, they could use silence pedagogy or other interventions to enhance engagement.
